# Associations between Dietary Patterns and Inflammatory Markers during Pregnancy: A Systematic Review

**DOI:** 10.3390/nu13030834

**Published:** 2021-03-04

**Authors:** Kuan-Lin Yeh, Amber Kautz, Barbara Lohse, Susan W. Groth

**Affiliations:** 1 School of Nursing, University of Rochester, Rochester, NY 14642, USA; 2 School of Medicine and Dentistry, University of Rochester, Rochester, NY 14623, USA; amber_kautz@urmc.rochester.edu; 3 Wegmans School of Health and Nutrition, Rochester Institute of Technology, Rochester, NY 14623, USA; balihst@rit.edu

**Keywords:** diet, dietary patterns, inflammation, inflammatory markers, pregnancy

## Abstract

Elevated inflammation in pregnancy has been associated with multiple adverse pregnancy outcomes and potentially an increased susceptibility to future chronic disease. How maternal dietary patterns influence systemic inflammation during pregnancy requires further investigation. The purpose of this review was to comprehensively evaluate studies that assessed dietary patterns and inflammatory markers during pregnancy. This review was guided by the Preferred Reporting Items for Systematic Review and Meta-Analyses. Included studies were sourced from EMBASE, PubMed, Web of Science, and Scopus and evaluated using The Quality Assessment Tool for Quantitative Studies. Inclusion criteria consisted of human studies published in English between January 2007 and May 2020 that addressed associations between dietary patterns and inflammatory markers during pregnancy. Studies focused on a single nutrient, supplementation, or combined interventions were excluded. A total of 17 studies were included. Despite some inconsistent findings, maternal diets characterized by a higher intake of animal protein and cholesterol and/or a lower intake of fiber were shown to be associated with certain pro-inflammatory markers (C-reactive protein (CRP), interleukin-6 (IL-6), tumor necrosis factor-α (TNF- α), IL-8, serum amyloid A (SAA), and glycoprotein acetylation (GlycA)). Future studies that explore a broader range of inflammatory markers in the pregnant population, reduce measurement errors, and ensure adequate statistical adjustment are warranted.

## 1. Introduction

An amplified inflammatory response during pregnancy has been linked to multiple pregnancy complications [1], such as preeclampsia [2], preterm delivery [3], depressive symptoms [4], and recurrent abortion [5]. Maternal inflammation also contributes to programming of fetal metabolic profiles and immune system through epigenetic modifications [6,7]. Systemic inflammation normally occurs during pregnancy [8]. Pro-inflammatory markers, such as interleukin-6 (IL-6), tumor necrosis factor-α (TNF- α) [9,10], and C-reactive protein (CRP) [11], increase significantly over the course of pregnancy. The immune response is particularly increased at the times of implantation and parturition [12,13,14] or during periods of excess maternal psychosocial stress [9]. High levels of IL-6 and TNF-α have been positively associated with gestational diabetes mellitus (GDM) [15]. Elevated CRP and IL-8 have been associated with an increased risk of preeclampsia [16]. The activation of inflammasomes, which induce interleukin 1β (IL-1β) secretion, has been linked to maternal obesity, preterm birth, and preeclampsia [13]. Pro-inflammatory cytokines (IL-6, TNF-α, IL-8, IL-1β) have been found to remain elevated or increase in the first month after childbirth [10]. Women with a history of preeclampsia and persistent inflammation during postpartum have an increased risk of cardiometabolic disease and neurodegenerative disorders later in life [17]. 

The immune response is activated as the body attempts to restore homeostasis following an irritant stimulus [18]. If the response is amplified, increased oxidative stress and vasoconstriction may result in adverse pregnancy outcomes [1]. Activated transcription factors (e.g., nuclear factor kappa-B (NF-κB)) and increased inflammatory cytokines, in turn, amplify inflammatory response and contribute to the development and progression of disease processes [18,19]. In the non-pregnant population, chronic inflammation has been shown to contribute to the development of autoimmune disorders [20], endothelial dysfunction [21], cardiometabolic disorders [20,22,23], and increased risk of cardiovascular morbidity and mortality [24]. Pharmacotherapy targeted at IL-1β [25] and TNF-α [24] has been shown to successfully improve cardiovascular and endothelial function, suggesting that controlling the inflammatory response is a potential target for research. 

Evidence from global studies has indicated that diet has a crucial role in the prevention of chronic disease and mortality [6,26]. Multiple nutrients and bioactive substances, such as vitamin E, affect the processes of intracellular signaling and the expression of genes involved in inflammation, with consequent reduction of pro-inflammatory cytokine synthesis [19]. For example, plant phenolic compounds enhance immune function [27] and reduce certain enzymes involved in the generation of reactive oxygen species [28]. Polyphenol antioxidants can modulate immune homeostasis and intracellular signaling by inactivating the NF-κB pathway, modulate mitogen-activated protein kinase (MAPK) [27,29], and suppress toll-like receptor (TLR) [28]. Omega-3 polyunsaturated fatty acid attenuates the activation of TLR4, thereby reducing the production of pro-inflammatory mediators [30]. Conversely, increased dietary exposure to saturated fatty acids and free fatty acids trigger the activation of the c-Jun N-terminal kinase (JNK) and IκB kinase-β (IKKβ) signaling pathways [31,32]. A high-fat diet has been linked to increased intestinal permeability (leaky gut), resulting in an altered gut microbiome [33] and increased lipopolysaccharide (LPS) (bacterial endotoxins) [34], which activate TLR4 [30]. A complex network of signaling pathways is involved in the interaction of dietary and microbial metabolites and the production of inflammatory mediators [18,19]. 

An increased interest in synergistic interactions among individual nutrients and, ultimately, dietary patterns has emerged [35]. Dietary patterns refer to foods and beverages that are habitually consumed with respect to their amounts, proportions, variety, combinations, and/or frequency [36]. In the non-pregnant population, high intake of fruits and vegetables, such as in the Nordic diet and Mediterranean diet, have been shown to reduce inflammation [37,38,39,40] and promote endothelial function [38] and cardiovascular health [37,41]. On the other hand, a high intake of saturated fats, added sugars, and simple carbohydrates have been linked to increased inflammatory markers (CRP/high sensitivity (hs)-CRP, IL-6) [42,43] and risk of hypertension [44] and cardiovascular disease [37,45]. Adherence to a Mediterranean diet may modulate low-grade inflammation in pregnancy [46]. A randomized clinical trial (RCT) using a combined lifestyle approach demonstrated the positive effects of carbohydrates on hs-CRP concentrations in late pregnancy [47]. Inflammation-targeted nutrition therapy may provide potential prophylaxis, but little is known about which dietary pattern influences systemic inflammation and which inflammatory markers are altered by diet during pregnancy. Therefore, the aim of this review is to synthesize the current empirical evidence concerning the relationship between dietary patterns and the inflammatory response and identify the inflammatory markers associated with dietary patterns during pregnancy.

## 2. Materials and Methods 

This review was guided by the Preferred Reporting Items for Systematic Review and Meta-Analyses (PRISMA). Articles were sourced from 4 databases, namely, EMBASE, PubMed, Web of Science, and Scopus. The literature search was undertaken in September 2018 and updated in May 2020. The search strategy was adapted for each database using keywords as follows: inflammation OR “inflammatory markers” AND pregnancy OR “pregnant women” AND diet OR “dietary patterns.” No restrictions or filters were applied to the search in the title, abstract, or keywords. Two reviewers (K.Y. and A.K.) independently performed the screening of titles and abstracts, followed by full-text assessment as needed. 

The screening process involved the following inclusion criteria: (1) pregnant women as the target population, (2) maternal dietary patterns as the predictor or independent variable, (3) peripheral blood markers of inflammation as the outcome or dependent variable, (4) emphasis on dietary patterns rather than single nutrients, (5) publication in a peer-reviewed journal between 2007 and 2020, (6) intervention or observational studies, (7) publication in English, and (8) human studies with full-text only. For multiple publications based on the same study, only the one with the largest sample size was included. Studies on supplementation or dietary exposure combined with other lifestyle interventions were excluded. The reference lists of included articles were carefully reviewed to identify any additional eligible studies. The screening process was conducted using Endnote and Abstrackr software. Data extraction included several key components (i.e., author, country, study design, participant characteristics, sample size, dietary pattern(s), reference period, dietary assessment tool, and the main findings related to the levels of inflammatory markers) that were verified by a second reviewer. All discrepancies were discussed, and a consensus was reached. 

The quality of included studies was independently assessed by 2 reviewers (K.Y. and B.L. or A.K.) using the “Quality Assessment Tool for Quantitative Studies” developed by the Effective Public Health Practice Project (EPHPP) [48]. This appraisal tool has a strong rating in methodology and has been widely used in public health research [48]. Studies were evaluated on 8 key components, namely, selection bias, study design, confounders accounted for, blinding, data collection methods, withdrawals and dropouts, intervention integrity, and analysis. If any of the essential information for the EPHPP quality assessment was absent, cited references were reviewed in detail. A global ranking of strong, moderate, or weak was determined on the basis of the first 6 criteria in accordance with the guideline recommendations [48]. Any discrepancies between the two reviewers’ data extraction and global ratings, the included articles, and its relevant reference were reassessed and discussed, and a consensus was reached. A third person was approached for the resolution of any unresolved discrepancies. 

## 3. Results

**Figure 1:**
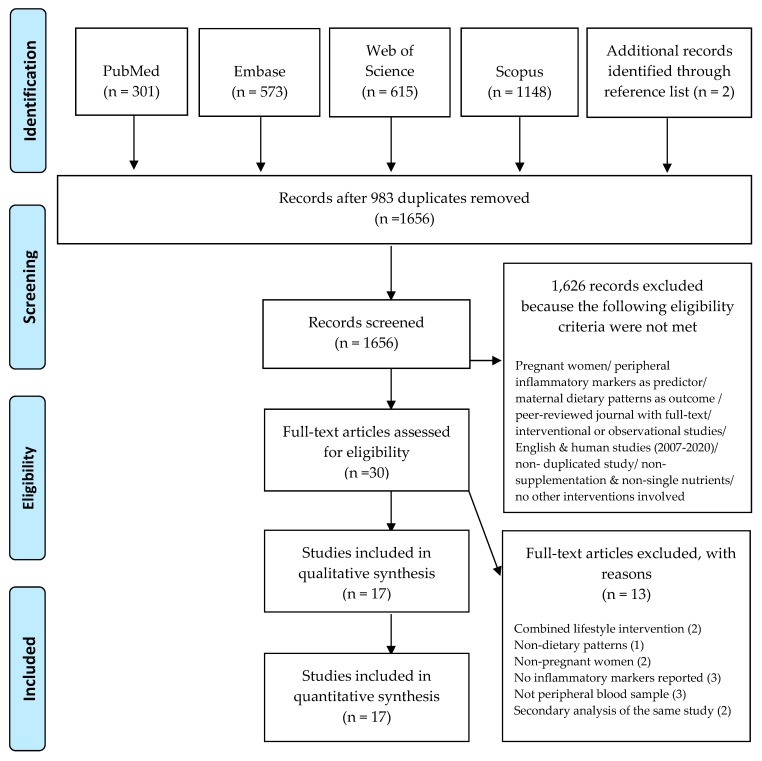
Literature was searched in September 2018 and updated in May 2020. The included studies were published from 2007 to May 2020.

A total of 2639 relevant articles from four databases were extracted, including two articles identified through a review of article reference lists. Of those, 983 duplicates were removed, and the titles and abstracts of the remaining 1656 studies were screened for their eligibility on the basis of inclusion and exclusion criteria. Following the screening, an additional 1626 articles that did not meet the inclusion criteria were excluded. Of the remaining 30 studies, 13 were determined ineligible on the basis of a full-text evaluation. A total of 17 articles were included in the present review. The detailed process of article selection based on the PRISMA guidelines is presented in Figure 1.

### 3.1. Study Characteristics

Characteristics of the included studies are outlined in Table 1. Included studies were conducted in 10 countries from 5 continents, namely, Europe, Australia, Asia, and South and North America. Six studies were from the United States [3,49,50,51,52,53], two were from Iran [54,55], and one each was from China [56], Lebanon [57], the United Kingdom [58], Norway [59], Denmark [60], Australia [61], Finland [62], Ireland [63], and Brazil [11]. Among the included studies, four studies were randomized controlled trials [54,58,59,61]. Sample sizes ranged from 32 [54] to 290 [61], and intervention duration ranged from four weeks [54] to five months [58]. Seven studies were prospective cohort designs [3,11,49,50,51,52,56] with the sample sizes ranging between 116 [11] to 1808 [52]. Five studies were cross-sectional [53,57,60,62,63], with the sample sizes ranging between 100 [62] to 621 [63], and one study was a case-control study with 135 participants [55].

Study samples were diverse and representative of a variety of pregnant populations. Seven studies presented the ethnicity distribution of their study sample [3,49,50,51,52,53,61]. Predominately represented ethnicities included Non-Hispanic white, Non-Hispanic black, and Hispanic. Participants were between 16 [51] and 45 years of age [55]. The health status of participants varied across studies, including pregnant women who were healthy [3,11,50,51,56,57,58,59,60], healthy but at high risk of GDM [61], healthy but at high risk of giving birth to an infant with macrosomia [63], non-diabetic and with a body mass index (BMI) 18.5 kg/m^2^ [52], overweight or obese [62], or who had a history of recurrent miscarriages [55] or GDM [54]. Two studies did not specify participants’ pre-existing health conditions as part of the enrollment criteria [49,53]. Nine studies described the education distribution of their study sample, indicating the majority of participants had achieved a high school degree or higher [11,49,50,51,52,53,55,61,62]. 

### 3.2. Dietary Patterns

The observational studies included a priori defined dietary patterns, such as the Mediterranean diet [57], low/high glycemic index (GI) diet, low/high glycemic load (GL) diet [3,11], and pro-/anti-inflammatory diets using the Dietary Inflammatory Index (DII) [50,51,52,53,55,56] or Energy-Adjusted Dietary Inflammatory Index (E-DII) [49]. Other dietary patterns were a posteriori derived through a statistical approach with an emphasis on the proportions of macronutrients [40,49,51], fiber [49,51], or cholesterol [51].

Dietary interventions following a priori*-*defined dietary patterns included the addition of salmon biweekly [58], a low-GI diet [61], an anti-atherogenic diet [59], and the Dietary Approaches to Stop Hypertension (DASH) diet [54]. Common characteristics of these diets included high amounts of fresh plant-based foods (e.g., fruits and vegetables and whole grains) and low intake of processed meats and animal fats [64,65]. These patterns are largely in accordance with the Global Action Plan for the Prevention and Control of Noncommunicable Disease [65]. 

### 3.3. Dietary Assessment

A variety of dietary assessment tools, including food frequency questionnaires (FFQ), 24-hour dietary recalls, and three-day food diaries/records, were utilized to measure dietary intake. FFQs were used in seven studies [11,49,52,55,57,58,60]. The number of items on the FFQs ranged from 100 [58] to 168 [55]. The reference periods of the FFQs varied from the previous 3 months [52,60,66], 3 to 6 months [49], or 12 months [67,68]. The questionnaires were specifically developed for assessing the Mediterranean diet [57], the Middle Eastern diet [69], the Brazilian diet [11], marine n-3 fatty acids intake [60], or the nutrient intake in pregnant Caucasian women [58]. Two were adapted from the Willett questionnaire [52,55]. One was developed by the University of Texas MD Anderson Cancer Center [49]. Six studies used 24-hour dietary recalls. Of these, two utilized a single recall [53,57], and four used repeated recalls collected consecutively for three days [56] or non-consecutively at two time-points [3], at three time-points [50,61], or every month during pregnancy [51]. Three-day food diaries/records were used in three studies [54,61,62]. 

Eight of the studies included only a single dietary measure to assess maternal dietary patterns in the first trimester [62], the second trimester [53,54,55,56,60], or both the first and second trimester [11,57]. A total of eight studies included repeated dietary measures during the first half of pregnancy [3,52], the second half of pregnancy [58,59,61], or throughout the entire pregnancy [49,50,51]. Dietary compliance was tracked in the intervention studies with a self-reported five-point Likert adherence scale [63], a fish consumption diary [58], multiple 24-hour dietary recalls [61], or a weighted dietary measure on a pre-determined day every week [59]. Notably, nearly half of the studies [3,50,51,56,57,60,62,63] did not include an assessment or estimation of dietary supplement intake, which is essential to estimate total nutrient intake.

### 3.4. Peripheral Markers of Inflammation

The most frequently exmined inflamatory markers in relation to diet were CRP [11,52,53,55,57,61]/hs-CRP [3,51,54,56,59,60,62] and cytokines (cell signaling molecules) including TNF-α [49,50,58,63], IL-6 [49,51,55,58,63], and IL-8 [58,60]. Less explored cytokines were IL-1β, IL-4, IL-17A, IL-12p70, interferon gamma (IFNγ) [49], hepatocyte growth factor (HGF), and monocyte chemotactic protein-1 (MCP-1)—a subfamily of cytokines [58]. Soluble cell adhesion molecules (sCAMS) included vascular adhesion molecule-1 (sVCAM-1), intercellular adhesion molecule-1 (sICAM-1), and E-selectin [58,59]. Other reported inflammatory markers were glycoprotein acetylation (GlycA; a novel low-grade inflammatory marker) [62], serum amyloid A (SAA; an acute-phase protein) [60], and nerve growth factor (NGF; neuroinflammatory markers) [58]. Blood specimens were drawn from pregnant women in a fasted state [11,54,56,57,58,61,62,63] or during glucose screening tests [3,52]. A variety of biochemical analysis techniques were used in detecting and quantifying inflammatory markers, including enzyme-linked immunosorbent assay (ELISA) [3,54,57,63], nephelometry [53], immunoturbidimetry [11,51,56], and multiplexed method [49,51,52,58,60].

### 3.5. Risk of Bias Assessment

The quality assessment of the included studies is displayed in Table 2. Strong ratings in selection bias were given to studies with participation rates above 80% [3,54]; weak ratings were given to studies with participation rates less than 60% [61,62]. Only RCTs were rated as strong in study design. Randomization was performed using a random number table [58,59,66] or computer software program [54,61,63,74]. For confounders, strong ratings were given to RCTs with groups balanced at baseline [54,58,59,61], the cross-sectional analysis of an RCT [63], and eight observational studies [3,11,49,50,51,52,53,60] that controlled for a number of possible confounding variables (e.g., age, BMI, ethnicity, education, income, parity, smoking). Moderate ratings were given to two observational studies that did not control for smoking status [55,56], which is a notable confounding variable. Two cross-sectional studies that controlled for less than 60% of confounders [57,62] were identified as weak. A few studies addressed anti-inflammatory medication use [62] and acute inflammatory conditions [11,50,55,57]. 

A double-blind design in the RCTs was considered strong [54], and a single-blind design moderate [58,59,61]. All observational studies, with the exception of the study using the data from National Health and Nutrition Examination Survey (NHANES) [53], were rated as weak for blinding. Strong ratings were given to the studies using standard assessment tools (e.g., Willett FFQ or 24-hour dietary recalls) [3,49,50,51,52,55,56,57,61,62,63]. Instruments with known validity or reliability are recognized to have good ratings in data collection methods per the EPHPP dictionary [75]. The study using one single 24-hour dietary recall for measuring dietary intake [53] was rated as moderate. The method of using predetermined days for dietary reporting, which is likely to be altered by social disability, was evaluated as weak [59]. Moderate ratings in withdrawals and dropouts were given to studies with a case–control design [55], a study completion rate of less than 80% [51,53,60], a lack of reporting the reasons for withdrawal [59], or only reporting the number of cases with completed data [3,50]. Overall, the majority of the included studies were classified as moderate methodological quality [3,11,49,50,51,52,55,56,58,59,60,61,63]. Two studies were classified as weak quality [57,62], and three studies were classified as strong quality [53,54,58].

### 3.6. Dietary Patterns and Inflammatory Markers

Dietary patterns described in the studies were not uniform and therefore were categorized on the basis of how the pattern was defined (a posteriori-derived vs. a priori-defined) along with the research design. The majority of observational studies (*n* = 10) indicated a statistically significant association (*p* < 0.05) between diet and inflammatory markers (CRP/hs-CRP, IL-6, IL-8, TNF-α, SAA, or GlycA). However, diet–inflammation associations were not found in the intervention studies.

#### 3.6.1. Proportions of Protein, Cholesterol, and/or Fiber

A posteriori-derived approach was used to examine the proportions of macronutrients among the observational studies. Dietary patterns characterized by higher cholesterol and protein intake were associated with increased hs-CRP in the first trimester [3]. Protein from animal sources was associated with higher hs-CRP and SAA in the third trimester [60]. Lower fat (especially saturated fatty acids) and higher fiber intake contributed to greater diversity and richness of the gut microbiome, which was correlated with lower GlycA but not hs-CRP in the first trimester [62]. Dietary intake of fiber was found to be inversely correlated with levels of CRP in the second trimester [57] and IL-8 in the third trimester [60]. 

#### 3.6.2. Anti-/Pro-Inflammatory Diet

In the observational studies, some inconsistencies were found among the studies of pro-inflammatory diets quantified by the DII (the higher the pro-inflammatory potential of diet, the higher the DII). DII was shown to be positively associated with levels of CRP [52]/hs-CRP [56] during the second trimester when repeated dietary measures were used. Conversely, a positive association between DII and hs-CRP in the second trimester was not found using a single 24-hour dietary recall [53]. The associations between DII and CRP/hs-CRP were not statistically significant in the first [55] or third [51] trimester. Certain cytokines were positively associated with DII, including IL-6 in the first [55] and second [51] trimesters and TNF-α across all three trimesters [50]. Notably, the association between DII and TNF-α was only observed in pregnant women with high levels of stress [50]. Inflammatory diets measured by the E-DII were not associated with circulating cytokines (TNF-α, IL-6, IL-1b, IL-4, IL-17A, IL-12p70, IFNγ) in early pregnancy [49].

#### 3.6.3. High/Low GI/GL Diet

With respect to carbohydrate content and associated glycemic burden, the results were mixed among the observational and intervention studies. In one cohort study, GI was positively associated with hs-CRP among healthy women with a BMI less than 25 kg/m^2^; dietary glycemic load (GL), which addresses both the quality and quantity of carbohydrates, was not associated with hs-CRP levels in the first trimester [3]. In contrast, a different cohort study indicated a non-significant association between GI and CRP, but an inverse association between GL and CRP in healthy women throughout pregnancy [11]. An intervention of low GI advice had no impact on levels of CRP in women with a high risk of GDM [61] nor on IL-6 and TNF-α in women at high risk for macrosomia [63] in the second half pregnancy.

#### 3.6.4. Mediterranean Diet, DASH Diet, Anti-Atherogenic Diet, and Omega-3 Fatty Acid-Enriched Diet

Findings from an observational study indicated a significant association between higher adherence to the Mediterranean diet and lower CRP levels in the second trimester [57]. However, an effect of diet on inflammation was not observed in the intervention studies focusing on an anti-atherogenic diet [59], a DASH diet [54], or omega-3 fatty acid-enriched diet [58]. Specifically, an intervention using the DASH diet had no significant effects on levels of hs-CRP in the second trimester [54]. An anti-atherogenic diet had no effect on levels of hs-CRP and sCAMS [59], and regular salmon intake had no effect on IL-8, IL-6, TNF- α, HGF, NGF, MCP-1, HGF, or sCAMS in the second half of pregnancy [58]. 

## 4. Discussion

This systematic review presents evidence from observational studies and controlled trials of relationships between maternal dietary patterns and inflammatory markers. Dietary patterns explored in this review included the pro-/anti-inflammatory diet, low GI/GL diet, Mediterranean diet, DASH diet, and an anti-atherogenic diet. Additionally, the macronutrient composition and regular salmon intake, which addressed the proportions or frequency of dietary components, were examined. Overall, about two-thirds of the studies with moderate quality in methodology per EPHPP showed associations between dietary patterns and pro-inflammatory markers; however, the three studies with strong methodology did not report significant associations, including one observational study [53] and two intervention studies [54,58]. 

### 4.1. Evidence from the Observational Studies

A dietary pattern with high animal protein, high cholesterol, and/or low fiber was significantly associated with higher levels of CRP [52,57,60]/hs-CRP [3,56] and SAA [60] during pregnancy. Lower fiber intake was associated with higher IL-8 [60] and reduced gut microbiota richness, which contributes to higher GlycA [62]. Interestingly, microbiota composition was not correlated to hs-CRP, suggesting a possible different mechanism of inflammation [62]. Diets categorized as low-DII [50,51,52,55,56] were associated with lower levels of pro-inflammatory markers in pregnancy. Dietary intake with less anti-inflammatory food parameters (e.g., fiber, vitamins, β-carotene, and flavones) and more pro-inflammatory food parameters (e.g., saturated fats, cholesterol) were associated with higher IL-6 [51,55] and TNF-α [50]. Notably, adherence to a Mediterranean diet was correlated with lower CRP [55].

Relationships between dietary GI/GL and inflammatory markers were inconsistent. Inflammation was found to be positively associated with GI [3] and negatively associated with high GL diets [11]. GI ranks carbohydrates according to their effect on blood glucose levels [76]. GL takes both the quality and total amount of carbohydrates into consideration and is a mathematical product of GI and the quantity of carbohydrates consumed [76]. An inverse association between GL, but not GI, and inflammatory markers [11] could be a function of multiple factors. First, the GI and GL values were derived from a FFQ measurement that consisted of broad food groupings [67], increasing the likelihood of misclassification and an underestimation of an association [76]. Second, this particular FFQ was shown to overestimate fruit intake [67], a systematic error that could lead to a biased finding [77]. Third, a low GL diet could be low in carbohydrates but high in saturated fat and protein [76], which are pro-inflammatory [78]. Compared to a high complex carbohydrate/low fat dietary pattern, a conventional diet low in carbohydrates and high in fat was associated with elevated inflammation during pregnancy [79]. Variations in individual insulin response, mixed meals that influence the glycemic response, and food processing techniques are other possible factors affecting GI/GL [76]. 

The association of DII with inflammatory markers was inconsistent across four studies [49,51,53,55]. Higher DII was associated with a higher concentration of CRP/hs-CRP in two studies [52,56] but not replicated in the DII studies that showed a statistically significant association between a pro-inflammatory diet and IL-6 [51,55]. One factor to consider is that IL-6 is an inducer of hepatic CRP synthesis in hepatocytes [80,81]. Given the mediating effects of IL-6 on the production of hs-CRP, the timing of inflammatory marker measurement could influence the results [51]. In addition, DII was associated with TNF-α only in the context of psychological stress. Among the DII studies, two containing notable measurement issues showed a non-significant association between DII and inflammatory markers [49,53]. One cross-sectional study using NHANES data was limited by its dietary measurement of a single 24-hour dietary recall [53] to represent usual dietary intake, increasing the risk of exposure misclassification [77]. Another study included a DII that was energy-adjusted to account for variation in the total energy consumption [49]. However, despite collecting multiple dietary assessments throughout pregnancy, blood samples were collected only at enrollment. Given the changing nature of dietary intake and inflammation during pregnancy, corresponding repeated measures are necessary to reduce measurement error. 

Measurement of dietary intake can be challenging. Therefore, the majority of the longitudinal studies used repeated measures of dietary assessments [3,49,50,51,52], enabling a better estimation of habitual intake [77]. To improve dietary estimates, differing dietary assessment tools can be used in combination [82]. For example, measurement error was reduced in one investigation of adherence to the Mediterranean diet by using both a FFQ and a 24-hour dietary recall [57]. Other factors that may have influenced associations between dietary patterns and maternal inflammatory markers include confounding factors, such as age, BMI [53,83], parity [11,83], and smoking status [83,84]. Nearly two-thirds (*n* = 8) of the included observational studies adjusted for known confounders. Among the observational studies with a full model adjustment, a significant association between dietary pattern and inflammation was reported [3,50,51,52,60]. However, information regarding the use of anti-inflammatory medication (e.g., nonsteroidal anti-inflammatory drugs) or the occurrence of acute infections (e.g., periodontitis, urinary tract infections) was frequently left out [85]. Additionally, genetic and microbiome profiles that may influence the inflammatory response to nutrients [85,86] were not considered. 

### 4.2. Evidence from the Intervention Studies

Causal effects of the low GI diet, DASH diet, anti-atherogenic diet, and regular salmon intake on inflammation during pregnancy were not evident in the examined RCTs. The lack of blinding in many of the studies, although a challenge in dietary intervention research [87], could lead to considerable expectation bias [88]. Unblinded participants could change their behaviors, and unblinded outcome assessors could unintentionally produce biased results, thereby inducing threats to internal validity [88].

The effect of dietary GI on CRP levels in pregnancy was not established [61]. Three-day food records were combined with 24-hour dietary recalls [61] to improve the accuracy of dietary measurement. However, the intervention and control diets were similarly healthy, which reduced variation in dietary exposure and the ability to detect differing effects of the interventions [89]. In addition, this GI study may have been underpowered given that inflammation was neither the primary nor secondary outcome [90]. Of note, the specification of the type of carbohydrate was lacking in this study, decreasing the interpretability of findings. A complex carbohydrate is mainly sourced from unprocessed and whole, plant-based foods [91], whereas simple carbohydrates are often found in highly processed foods [91]. Whole, plant-based foods are generally accepted as anti-inflammatory, whereas ultra-processed foods are considered pro-inflammatory [92].

The DASH diet did not have an effect on hs-CRP concentrations in pregnant women. However, the sample size of this RCT was small (*n* = 32), and groups reported similar amounts of energy and protein intake and high amounts of vegetable and fruit intake over the 4-week study period [54]. Additionally, associations between the omega-3 fatty acid-enriched diet and plasma inflammatory markers were not established [58]. A study that focused on the effects of routine salmon intake on cytokines using combined dietary assessments (a FFQ and a seafood diary [66]) indicated a non-significant result. The authors concluded that omega-3 intake may have been too low (<2 g/day) to reduce inflammation [58]. 

The anti-atherogenic diet did not have an effect on the levels of hs-CRP and sCAMS during pregnancy [59]. A reduced intake of cholesterol and saturated fat did not have a lowering effect on inflammation, which is contrary to the results of the observational study [3]. This discrepancy may be related to the methodological approach that included a weekly weighted dietary intake on a pre-determined day throughout pregnancy [59]. The predictability of this approach in the intervention study could have influenced participants’ dietary behaviors and compliance on the days of reporting their dietary intake. Furthermore, a type II error could have occurred, as a power calculation was not conducted for the outcome of inflammation [59]. 

### 4.3. Anti-Inflammatory Markers and Inflammation Resolution

Few studies in this review addressed anti-inflammatory cytokines in relation to dietary patterns. Only IL-4 [49] and HGF [58] were examined. Specialized pro-resolving mediators (SPMs), which are metabolites of omega-3 fatty acids responsible for resolving inflammation [93], were not explored. Current understanding of the anti-inflammatory properties of omega-3 fatty acids includes not only inhibition of pro-inflammatory signaling molecules but also the involvement of pro-resolving mediators to accelerate inflammation resolution [94,95]. Researchers have found that deficiencies in dietary omega-3 fatty acids may result in insufficient precursors of pro-resolving mediators, leading to prolonged inflammation [94]. An anti-inflammatory approach [25] and pro-resolving mediators [95] are emerging therapeutic targets for disease prevention in human research. To date, SPMs have not been explored in studies focusing on maternal dietary patterns. Pro-resolving mediators have only been explored in RCTs that focus on maternal dietary supplementation [96,97].

### 4.4. Strengths and Limitations

To the best of our knowledge, this is the first systematic review investigating dietary patterns and their association with inflammatory markers during pregnancy. The included studies were conducted in different pregnant populations across the globe, increasing the generalizability of findings. However, the associations between dietary patterns and inflammatory markers during pregnancy must be interpreted with caution for three main reasons. First, dietary assessment is a non-exact science and involves measurement problems. Second, variability in study design and rigor are evident, and third, types of dietary patterns and a lack of exactness in their description contribute to a less cohesive finding. Circulating cytokines (e.g., IL-6, IL-8, IL-1β, TNF-α) or CRP alone are non-specific inflammatory markers [90], and a consensus has not yet been reached for which markers are most pertinent or reflective of phase or acuity of inflammation in pregnancy [81]. Additionally, the exclusion of studies published in other languages limits the inclusion of findings reported by non-English speaking populations. Finally, a meta-analysis could not be conducted because of the methodological and statistical heterogeneity of the included studies.

### 4.5. Implications for Clinical Practice and Research

The findings of this review highlight the potential role of maternal dietary patterns to shape the inflammatory response during pregnancy. Consuming certain dietary patterns (e.g., high intake of saturated fats and low intake of fruits and vegetables) may contribute to higher concentrations of inflammatory markers in pregnancy. Anti-inflammatory dietary patterns correspond to healthy patterns of eating, which is consistent with adherence to the current dietary recommendations. Future studies are needed to examine the changes of anti-inflammatory markers and pro-resolving mediators in relation to maternal dietary patterns to improve understanding of dietary effects in the context of pregnancy. In the research design phase, researchers can consider the adjustment of multiple confounding variables, such as psychosocial and lifestyle factors and the use of anti-inflammatory medication. Additional longitudinal studies with robust methodology and large sample sizes are needed to investigate the associations between dietary patterns and inflammatory response among pregnant women. 

## 5. Conclusions

Findings from the observational studies, although inconsistent, suggest that dietary patterns may be associated with pro-inflammatory markers, such as CRP, IL-6, IL-8, TNF-α, SAA, and GlycA, during pregnancy. Maternal dietary patterns characterized by high intakes of animal protein and cholesterol, and/or low intakes of fiber were associated with higher inflammatory status. However, all of the included intervention studies reported no dietary effects on inflammation. The most commonly studied inflammatory marker in pregnancy is CRP, but only 6 out of 13 studies showed any relationship of CRP with a dietary pattern. Discrepancies in the findings among the assessed studies may be partly because of measurement errors and timing of data collection. These discrepancies are reflective of the complexities and unknown ramifications of dietary effects on inflammation during pregnancy. Future longitudinal studies investigating a broader range of inflammation-related biomarkers throughout pregnancy, coupled with rigorous designs, are warranted.

## Figures and Tables

**Table 1:** Characteristics of the studies examining the relationship between diet and inflammatory biomarkers.

Study Design	Reference -Country/Sample Size		Maternal Age (Years Old)	Health Status and Gestational Age at Enrollment	Dietary Exposure /Control	Dietary Assessment Tool (FFQ Reference Period) Time of Dietary Data Collection	Time of Blood Collection	Examined Inflammatory Markers	Main Findings
Prospective cohort study	de Oliveira et al., 2015 -Brazil [11]	115	20–40	Healthy; 5–13 weeks of gestation	Glycemic load (GL) ≥ median /Glycemic load (GL) < median	A 73-item FFQ (a 12-month reference period) [67] collected at weeks 20–26 of gestation.	(Fasting) blood sample at 3 timepoints (weeks 5–13, 20–26, 30–36 of gestation)	CRP	Dietary glycemic load was negatively associated with CRP concentrations (β = –0.203; 95% CI, –0.380 to –0.026, *p* = 0.025).
	Lindsay et al., 2018 -USA [50]	202	>18	Healthy during the first or early second trimester	Anti-/pro- inflammatory diet	Interviewer administered 24-h dietary recalls collected at mean week 12.9 ± 1.7, 20.5 ± 1.4, and 30.4 ± 1.4 of gestation.	Day 6 of each assessment period at 10–12 weeks, 20–22 weeks, 30–32 weeks of gestation	TNF-α	The DII scores ranged from –4.3 to 3.7 using 32 food parameters. DII was positively associated with TNF-α (β = 0.093, 95% CI: 0.023–0.163, *p* = 0.010).
	McCullough et al., 2017 -USA [49]	1057	≥18	Ethnically diverse; <12 weeks of gestation	Anti-/pro- inflammatory diet	FFQ (a 6-month reference period) during peri-conceptional stage) at 3-time points: enrollment (median ~ 12 weeks), the second trimester (diet in the first trimester), between 36 weeks of gestation to delivery (diet in the last 2 trimesters), and at delivery as needed.	The blood sample was taken before 12 weeks of gestation	INFγ, IL-12, IL-17A, IL-1β, IL-4, IL-6, and TNF-α	The E-DII scores ranged from –5.00 to 4.96 using 27 food parameters. The correlation between maternal E-DII and circulating cytokines was not significant (*p* > 0.05).
	Moore et al., 2018 -USA [51]	511	≥16 (28 ± 6)	Healthy singleton pregnancy; <24 weeks of gestation	Anti-/pro- inflammatory diet	One ASA24 per month. At least one per participant. Visits in early pregnancy (median 17 weeks of gestation), mid-pregnancy (median 27 weeks of gestation), and at delivery (median 1 day after delivery)	Blood sample at 27 weeks of gestation	IL-6, hs-CRP	The DII scores ranged from –4.4 to 4.0 using 28 food parameters. For each 1 unit increase in the DII, a 0.12 mg/L increase in IL-6 levels was detected at 27 weeks of gestation (95% CI, 0.01–0.24; *p* = 0.03). No association was observed with hs-CRP (*p* = 0.27).
Study Design	Reference -Country/Sample Size		Maternal Age (Years Old)	Health Status and Gestational Age at Enrollment	Dietary Exposure /Control	Dietary Assessment Tool (FFQ Reference Period) Time of Dietary Data Collection	Time of Blood Collection	Examined Inflammatory Markers	Main Findings
Prospective cohort study	Scholl et al., 2011 -US [3]	520	Teenage and 19–32 [70]	Healthy; <20 weeks of gestation	The proportion of multiple dietary components	Two 24 dietary recalls at entry into prenatal care and at weeks 20–28 of gestation	(GCT) at entry into prenatal care	hs-CRP	Higher intakes of protein (*p* = 0.002) and cholesterol (*p* = 0.0016) with a lower intake of carbohydrate (*p* = 0.023), as well as a higher dietary glycemic index (*p* = 0.013) were associated with increased hs-CRP. only among lean gravidae (BMI <25 kg/m^2^).
	Sen et al., 2016 -USA [52]	1808	32.2 ± 5.0	Non-diabetic at median 9.9 weeks of gestation	Anti-/pro- inflammatory diet	A 146-item FFQ and 33 items for a supplement intake assessment [71] (last menstrual period for the first trimester or previous three months during the second trimester).	(GCT) at weeks 22–31 of gestation	CRP	The DII scores ranged from –5.4 to 3.7 using 28 food parameters. Higher DII was associated with higher plasma CRP in the second trimester (β = 0.08, 95% CI: 0.02–0.14).
	Yang et al., 2020 -China [56]	307	28.5 ± 3.4	Healthy with normal BMI; 16–20 weeks of gestation	Anti-/pro- inflammatory diet	Dietary recall for three consecutive days since the date of enrollment around 16–20 weeks of pregnancy.	(Fasting) after dietary assessment in the second trimester	hs-CRP	The odds of having high levels of hs-CRP in pro-inflammatory diet group were 1.89 times greater than the odds of having high levels of hs-CRP in the anti-inflammatory diet group (95% CI: 1.05, 3.42, *p* = 0.043).
Randomized controlled clinical trial	Asemi et al., 2013 -Iran [54]	32	18–40	GDM; 24–28 weeks of gestation	DASH /based on recommended acceptable dietary intake for GDM	Three-day food diaries throughout 4-week intervention period (24–28 weeks of gestation).	(Fasting) blood sample at 24–28 weeks of gestation and after 4-week intervention period	hs-CRP	Difference in mean change of serum hs-CRP between DASH and control diet was not significant (*p* > 0.05).
Study Design	Reference -Country/Sample Size		Maternal Age (Years Old)	Health Status and Gestational Age at Enrollment	Dietary Exposure /Control	Dietary Assessment Tool (FFQ Reference Period) Time of Dietary Data Collection	Time of Blood Collection	Examined Inflammatory Markers	Main Findings
Randomized controlled clinical trial	Garcia-Rodriguez et al., 2012 -United Kingdom [58]	123	18–40	Healthy; <19 weeks of gestation	Twice a week (150 g/portion) of salmon /habitual diet low in oily fish	A 100-item FFQ (a 12-week reference period) [66] collected at weeks 19/20 and 34 of gestation; a diary between week 20 of gestation to delivery.	(Fasting) blood sample at week 20, weeks 32–34 of gestation and at week 38 of gestation	IL-8, IL-6, TNF-α, HGF, NGF, MCP-1 sCAMs (E-selectin, ICAM-1, VCAM-1)	Inflammatory and vascular homeostasis biomarkers were not affected by the intake of farmed salmon (*p* > 0.05).
	Khoury et al., 2007 -Norway [59]	290	21–38	Healthy, non-smoking; 17–20 weeks of gestation	Anti-atherogenic diet (low in saturated fat and cholesterol) /usual diet	Weighed recordings of intake from baseline weeks 17–20 to week 36 of gestation.	Blood sample at weeks 17–20, week 24, week 30, and week 36 of gestation	hs-CRP, sCAMs (sVCAM-1, sICAM-1 and E-selectin)	None of the biomarkers were influenced by the intervention (low saturated fat/low cholesterol diet) compared to the control group (usual diet) (*p* > 0.05).
	Markovic et al., 2016 (GI baby3) -Australia [61]	139	>18	At high risk of GDM; 12–20 weeks of gestation	Low glycemic index (LGI) /high-fiber, moderate GI diet	Two 3-day food records collected at weeks 14–20 and 36 of gestation and three 24-hour dietary recalls as dietary compliance measure collected at weeks 18–24, 22–28, 26–32 of gestation; five dietary consultations from weeks 14–20 through 34–36 of gestation.	(Fasting) at mean week 17.4 ± 2 and at week 36 of gestation	CRP	Difference in CRP between the low glycemic diet group and high fiber group was not significant at the end of the intervention period (*p* > 0.05).
Cross-sectional	Hrolfsdottir ^ et al., 2016 -Denmark [60]	671	29 ± 4	Healthy; 283 ± 11 days of gestation	Protein intake and distribution between animal and plant sources	A 3-item self-administered FFQ combined with an interview (a 3-month reference period) [72] collected at week 30 of gestation.	Blood sample at week 30 of gestation	hs-CRP, SAA, IL-6, IL-8, IL-1β, TNF-α	Women in the highest compared to the lowest quintile of animal protein intake had 25% (95% CI: 2–53, *p* = 0.004) higher hsCRP concentrations. A similar pattern was observed for SAA (23%, 95% CI: 4–47, *p* = 0.003). Fiber intake was inversely associated with IL-8 (–24%, 95% CI: –37 to –9, *p* = 0.028).
Study Design	Reference -Country/Sample Size		Maternal Age (Years Old)	Health Status and Gestational Age at Enrollment	Dietary Exposure /Control	Dietary Assessment Tool (FFQ Reference Period) Time of Dietary Data Collection	Time of Blood Collection	Examined Inflammatory Markers	Main Findings
Cross-sectional	Papazian et al., 2019 -Lebanon [57]	100	18–40	Healthy singleton pregnancy; 14–27 weeks of gestation	Mediterranean diet adherence	A 157-item FFQ (12 categories of food groups; unspecified reference period) and one 24-hour recall collected between 14 and 27 weeks of gestation.	(Fasting) blood collection in the second trimester of pregnancy	CRP	Higher Mediterranean diet score was associated with lower CRP levels. MFP (OR: 0.90, 95% CI: 0.82–0.99, *p* = 0.03); MDS (OR: 0.88, 95% CI: 0.78–0.99, *p* = 0.04); Med Diet Score (OR: 0.88, 95% CI: 0.80–0.98, *p* = 0.02); SMDQ (OR: 0.91, 95%CI: 0.83–0.99, *p* = 0.04).
	Roytio et al. #, 2017 -Finland [62]	100	30.1 ± 4.7	Overweight and obese; ≤17 weeks of gestation	Three groups: low fiber/moderate fat ; high fiber/moderate fat; low fiber/high fat	One 3-day food diary per participant within the week before the study visit.	(Fasting) at mean week 13.3 ± 2.4 of gestation	hs-CRP, GlycA	Recommended dietary intake (DRI) of total fat and fiber was associated with lower levels of GlycA. Correlations between fiber total and GlycA were significant (*r* = 0.316, *p* = 0.01). No association was found between microbiota or intakes of nutrients and hs-CRP (*p* > 0.05).
	Shin et al., 2017 -USA [53]	561	20–44 [73]	NHANES samples at mean 5.35 ± 0.4 months of gestation	Anti-/pro- inflammatory diet	Single 24-hour dietary recall in the second trimester.	Blood sample in the second trimester	CRP	The DII scores ranged from –4.98 to 4.14 using 27 food parameters. DII was not associated with CRP (*p* > 0.05). Women who were obese before pregnancy had increased odds for being in the highest tertile of the DII and highest tertile of CRP concentration compared to women with normal weight.
Study Design	Reference -Country/Sample Size		Maternal Age (Years Old)	Health Status and Gestational Age at Enrollment	Dietary Exposure /Control	Dietary Assessment Tool (FFQ Reference Period) Time of Dietary Data Collection	Time of Blood Collection	Examined Inflammatory Markers	Main Findings
Cross-sectional	Walsh et al. *, 2014 -Ireland [63]	621	≥18	Secondigravid whose baby had macrosomia (birth weight > 4000 g) [74]	Low glycemic index (LGI) advice /not receiving LGI advice	A five-point Likert-type scale as an adherence measure at week 34 of gestation.	(Fasting) blood sample at mean week 13.8 ± 2.4 and 28 weeks of gestation	TNF-α, IL-6	Differences between those who did and those who did not receive low-GI dietary advice were not significant with respect to the concentrations of serum TNF-α or IL-6 in early pregnancy (prior to the intervention) or at 28 weeks of gestation (*p* > 0.05).
Case-control	Vahid et al., 2017 -Iran [55]	135	20–45	With a history of 3 or more miscarriages after week 20 of gestation	Anti-/pro- inflammatory diet	A 168-item FFQ (a 12-month reference period [68]).	Unspecified	IL-6, CRP	The DII scores ranged from –0.50 to 2.70 using 31 food parameters. For every 1 unit increase in DII, a corresponding increase in IL-6 by 0.15 pg/mL was detected (95% CI: <0.01–0.28, *p* = 0.04); no significant association was observed with CRP (*p* = 0.22).

Note: ASA24, Automated Self-Administered 24-hour Dietary Recall; BMI, body mass index; DII, Dietary Inflammatory Index; DRI, dietary reference intakes; E-DII, Energy-Adjusted Dietary Inflammatory Index; FFQ, Food Frequency Questionnaire; GCT, glucose challenge test; GlycA, glycoprotein acetylation; HGF, hepatocyte growth factor (cytokine); sICAM-1, soluble intercellular adhesion molecule-1; IFNγ, interferon gamma (cytokine); IL-6, interleukin-6; IL-1β, interleukin-1β; MCP-1, monocyte chemotactic protein-1 (chemokines); MDS, Mediterranean Diet Score; MDScale, Mediterranean Diet Scale; MedDietScale, the Mediterranean Diet Score; MFP, Mediterranean Food Pattern; NGF, nerve growth factor; NHANES, The National Health and Nutrition Examination Survey; SAA, serum amyloid A; sCAMs, soluble cell adhesion molecules; SMQD, Short Mediterranean Diet Questionnaire; TNF-α, tumor necrosis factor-α; sVCAM-1, soluble vascular adhesion molecule-1. ^ Cross-sectional analysis of a cohort study. # An exploratory analysis of an observational study. * Secondary analysis of a randomized controlled trial.

**Table 2:** Quality of the included studies assessed by the quality assessment tool for quantitative studies.

Study Design	First Author, Year	Selection Bias	Study Design	Confounders	Blinding	Data Collection Methods	Withdrawals/ Dropouts	Global Rating
Prospective cohort studies	de Oliveira et al., 2015 [11]	m	m	s	w	m	s	Moderate
	Lindsay et al., 2018 [50]	m	m	s	w	s	m	Moderate
	McCullough et al., 2017 [49]	m	m	s	w	s	s	Moderate
	Moore et al., 2018 [51]	m	m	s	w	s	m	Moderate
	Scholl et al., 2011 [3]	s	m	s	w	s	m	Moderate
	Sen et al., 2016 [52]	m	m	s	w	s	s	Moderate
	Yang et al., 2020 [56]	m	m	m	w	s	s	Moderate
Intervention studies	Asemi et al., 2013 [54]	s	s	s	s	s	s	Strong
	Garcia-Rodriguez et al., 2012 [58]	m	s	s	m	m	s	Strong
	Khoury et al., 2007 [59]	m	s	s	m	w	m	Moderate
	Markovic et al., 2016 [61]	w	s	s	m	s	s	Moderate
Cross-sectional studies	Hrolfsdottir ^ et al., 2016 [60]	m	m	s	w	m	m	Moderate
	Papazian et al., 2019 [57]	m	m	w	w	s	s	Weak
	Roytio # et al., 2017 [62]	w	m	w	w	s	s	Weak
	Shin et al., 2017 [53]	m	m	s	s	m	m	Strong
	Walsh * et al., 2014 [63]	m	m	s	w	s	s	Moderate
Case–control studies	Vahid et al., 2017 [55]	m	m	m	w	s	m	Moderate

*Notes:* Global rating (criteria): strong (no weak rating), moderate (one weak rating), and weak (two or more weak rating) [65]. ^ Cross-sectional analysis of a cohort study. # An exploratory analysis of an observational study. * Secondary analysis of a randomized controlled trial.

## Data Availability

Not applicable.
